# Electrochemical HOCl Production Modeling for an Electrochemical Catheter

**DOI:** 10.1149/1945-7111/ad8aee

**Published:** 2024-11-06

**Authors:** Dilara Ozdemir, Derek Fleming, Cristian Picioreanu, Robin Patel, Haluk Beyenal

**Affiliations:** 1 The Gene and Linda Voiland School of Chemical Engineering and Bioengineering, Washington State University, Pullman, Washington, United States of America; 2 Division of Clinical Microbiology, Department of Laboratory Medicine and Pathology, Mayo Clinic, Rochester, Minnesota, United States of America; 3 Water Desalination and Reuse Center (WDRC), Biological and Environmental Science and Engineering Division (BESE), King Abdullah University of Science and Technology (KAUST), Thuwal, Saudi Arabia; 4 Division of Public Health, Infectious Diseases, and Occupational Medicine, Department of Medicine, Mayo Clinic, Rochester, Minnesota, United States of America

**Keywords:** HOCl, electrochemical catheter, modeling, COMSOL

## Abstract

Hypochlorous acid (HOCl) is a strong oxidizing agent that damages cells by interacting with lipids, nucleic acids, sulfur-containing amino acids, and membrane components. It is an endogenous substance produced by the immune system to protect mammals from pathogens. Previously, we developed an HOCl-generating electrochemical catheter (e-catheter) and demonstrated its ability to prevent central line-associated bloodstream infections. The e-catheter is an electrochemical system consisting of two parts - an e-hub and a tube. Working, counter, and reference electrodes are placed in the e-hub, which contains 0.9% NaCl as an electrolyte. Although a prototype of this device has shown activity against pathogens, it is helpful to understand the factors influencing associated electrochemical/chemical processes to optimize design and efficacy. A mathematical model could predict factors influencing HOCl generation and distribution in the catheter and could aid in optimizing these devices. Here, we developed an Electrochemical Hypochlorous Acid Production (EHAP) model to predict factors influencing electrochemical generation and distribution of HOCl in e-catheters, including polarization time, diffusion of HOCl into the e-catheter, operational voltage, working electrode length, and surface area.

Intravascular catheters are healthcare devices used in hospitals, long-term care and outpatient facilities, and in patient’s homes. Peripheral lines are inserted in a peripheral vein and are typically used for short durations. Central venous catheters (CVCs) are inserted in large central veins (e.g., in the neck, chest, or groin), with vascular access points closer to the heart, and are typically used for long durations. It is estimated that CVCs are in place in nearly 8% of hospitalized patients in the United States,^1,2^ most often in the care of critically ill and cancer patients, for long-term administration of fluids and medications, and ease of access for repetitive blood draws, and are also used in outpatients for long-term medication or parenteral nutrition delivery, and hemodialysis.^3^

Intravascular catheters, and particularly CVCs, are prone to microbial colonization and subsequent systemic infection, as they provide direct pathogen access to the bloodstream. Central line associated bloodstream infections (CLABSIs) are defined as laboratory-confirmed bloodstream infections developing after 48 h of central line placement, originating from microbial colonization of catheters. These infections are stereotypically biofilm-associated,^4^ with colonizing pathogens attaching to catheters and secreting a self-synthesized extracellular matrix comprised of exopolysaccharides, extracellular DNA, lipids, and/or other molecules. It has been estimated that 250,000 CLABSIs occur globally each year,^5^ with an associated mortality rate of 25%.^6^ In the United States alone, nearly 40,000 CLABSIs are reported each year, at an annual cost of more than a billion dollars.^7–10^

CLABSIs occur via two routes - extraluminal colonization of the catheter tip at the insertion site of short-term venous catheters, and intraluminal colonization of long-term CVCs, mainly from the hub, where pathogens may be introduced directly into the lumen (catheter tube) via improper handling.^11^ Extraluminal infection risk may be mitigated, to some extent, by strict adherence to aseptic technique and reduction of pathogens at the skin interface, such as by using chlorhexidine-impregnated dressings.^12–14^ Prevention for intraluminal infections is more difficult; colonization can reach 40% for CVCs in place for more than 30 d.^15–18^ This poses a particular challenge for management of CLABSIs. Prevention and eradication is further complicated due to the frequent presence of multidrug-resistant organisms and biofilm formation.^19^ After CLABSI occurs, removal of the central vascular catheter is needed in many cases.^20^ However, the repetitive replacement of CVCs is not recommended.^21^ Thus, innovative approaches are needed for broad spectrum CLABSI prevention that avoids antimicrobial resistance, toxicity, and interference with drug delivery.

Several strategies to prevent intraluminal colonization of CVCs have been developed, including antimicrobial agent locks and antimicrobial-impregnated catheter lumens. Although antimicrobial locks have reduced CLABSI rates, this strategy is prone to complications, including antimicrobial resistance, allergic reactions, catheter damage, and potentially host toxicity due to the high concentrations of agents needed.^22,23^ Largely because of these factors, antimicrobial locks are infrequently used for CLABSI prevention in clinical practice. Antimicrobial impregnated catheters have also shown variable effects against CLABSI, but are not regularly used.^24–29^

A new approach, the integration of electrochemical technologies for hypochlorous acid (HOCl) production within intravascular catheters has emerged as a promising strategy to combat CLABSI. HOCl is a biocidal reactive oxygen species (ROS), naturally produced by phagocytes as part of the primary immune response,^30,31^ that exhibits broad spectrum activity against bacterial, fungal, and viral pathogens,^32–34^ with no evidence of resistance development.^34,35^ HOCl is used in industrial sectors, hospitals and households as a disinfectant. Safe handling and storage of HOCl may be challenging since high concentrations can have adverse effects on human health and environment.^36,37^ Future strategies for replacing conventional biocides with green biocides are needed due to their environmentally acceptable aspects. In situ electrochemically biocide generation is a way of producing green biocides due to mitigation of storage and transportation issues.^38^ In situ electrochemically generated HOCl can be used in biomedical applications, such as in the prevention of CLABSI infections.

In a previous study, HOCl generation from electrolysis of saline (0.9% NaCl, used clinically to flush intravascular catheters^39^) was studied for infection prevention in a prototype catheter system.^40^ This method is similar to antimicrobial lock therapy, aiming to sterilize the catheter by introducing an antimicrobial agent (HOCl) to the catheter lumen. However, antimicrobial/antibiotic lock therapy may fall short in providing desired concentrations and exposure times of the antimicrobial agent.^40^ In contrast, an electrochemical catheter (e-catheter) supplies continuous, low concentrations of HOCl directly into the catheter lumen to prevent colonization, biofilm growth, and subsequent bloodstream infection. We have shown that HOCl-generating e-catheters prevented bacterial catheter infection in vitro.^40^ In other studies, HOCl showed activity against both bacteria and fungi.^32–34^ Therefore, HOCl generation in a catheter system could be a suitable method for infection prevention.

Understanding HOCl generation and its dynamics in electrochemical systems like e-catheters is lacking, despite earlier research examining its antimicrobial activities. To maximize e-catheter efficacy, prediction of how HOCl concentrations change over time, how HOCl diffuses into the lumen, how electrode potentials influence HOCl concentrations, and how to manipulate HOCl generation for different purposes, is needed. This can be addressed experimentally. However, mathematical modeling is a quick and easy way to answer these questions. In addition, mathematical models can address how dissociation, degradation, and annihilation reactions affect HOCl concentrations. Another advantage of modeling is that simulations are not restricted by the practical detection limits of physical instruments; any concentration value can be simulated with no lower limit and an upper limit of 100% Faradaic efficiency (i.e., all electrical current is used exclusively for the intended reaction with no losses to side reactions or other processes).

Our goal was to develop a mathematical model to predict HOCl concentrations and dynamics in e-catheter systems and identify factors controlling them. Such knowledge can be used for e-catheter design for infection prevention by highlighting electrochemical aspects while also considering the medical context of CLABSI. We note that other studies have reported mathematical modeling of electrochemical HOCl generation from NaCl solutions, focusing on effects of diffusion, migration, and convection on mass transport of reacting species;^41–45^ these models focus on the effect of initial NaCl concentration using ion exchange membranes, catalytic reactions, and current efficiency. None of these models is applicable to e-catheter systems.

In this study, an Electrochemical Hypochlorous Acid Production (EHAP) model was developed to characterize HOCl generation by e-catheters. COMSOL Multiphysics^®^ mathematical model analysis was used to simulate continuous generation of HOCl by an e-catheter. The EHAP model, which can compute concentration profiles of HOCl, ClO^−^, ClO_2_^−^, ClO_3_^2−^, O_2_, H^+^, H_2_, OH^−^, Cl^−^, and Na^+^, was used to predict physical and chemical processes occurring during electrochemical production of HOCl. The solution in the e-catheter was considered stagnant, with transport processes expected to be controlled by diffusion. Further, the model simulated HOCl generation and diffusion under varying conditions, including polarization time, wait time after polarization, electrode potential, working electrode (WE) length, and surface area.

## Methods

### Electrochemical catheter and model geometry

**Figure 1.**
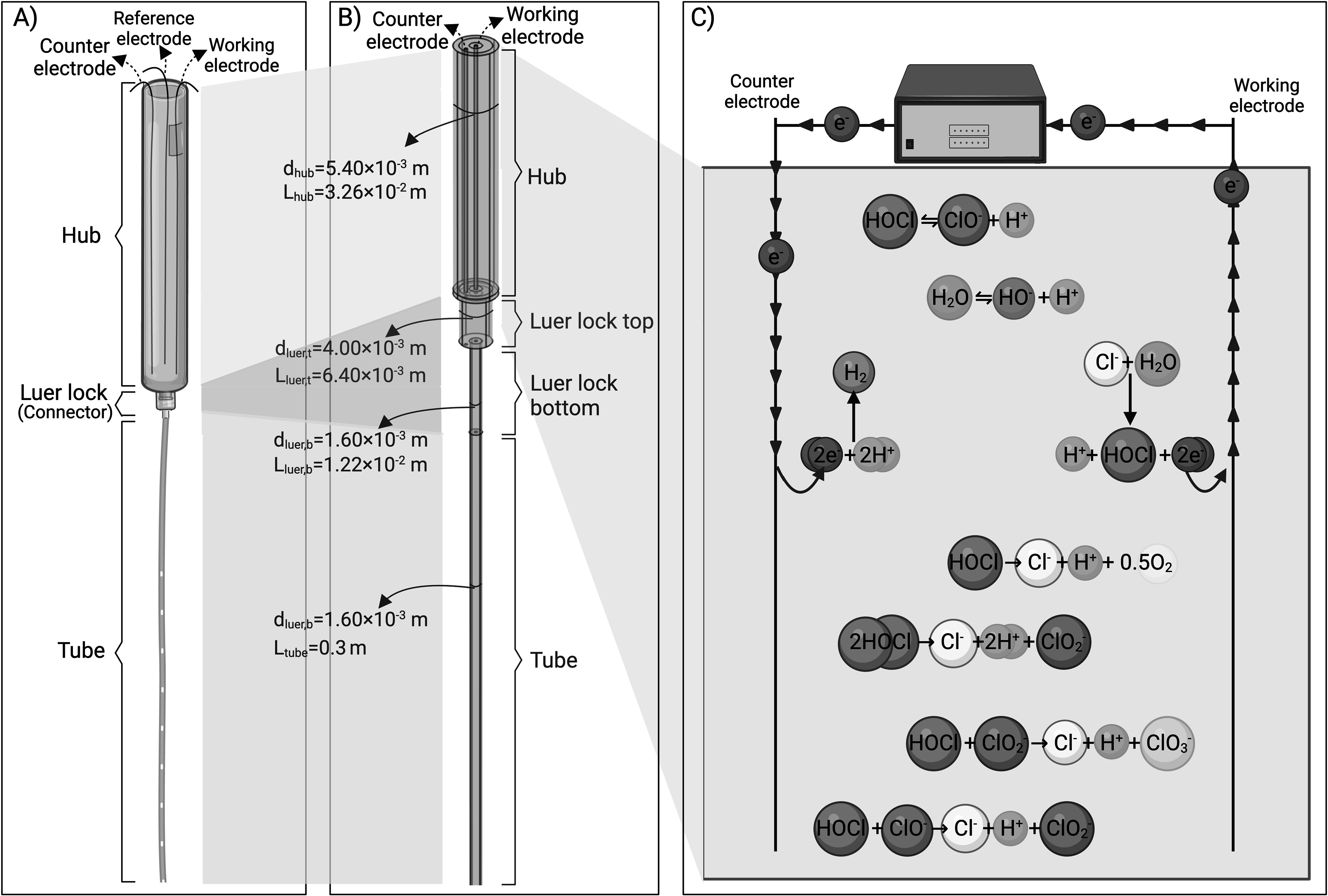
Illustration of (A) e-catheter, (B) COMSOL Multiphysics® 3D model geometry of the e-catheter. The entire e-catheter is termed the “e-catheter system” (L_e-catheter_: 0.3512 m) and (C) reactions occur in the e-catheter (created with BioRender.com).

A schematic representation of an e-catheter filled with 0.9% NaCl (saline) solution as electrolyte is presented in Fig. 1A. From top to bottom, the e-catheter has a hub, Luer locks (connectors), and a tube. The hub has a diameter of 5.4 mm and length of 32 mm. The Luer lock acts as a connector between the hub and tube and has two parts, a top with a diameter of 4 mm and length of 6.4 mm, and a bottom with a diameter of 1.6 mm and length of 12.2 mm. The tube has a diameter of 1.6 mm and a length of 0.3 m. Titanium or gold wires can be selected as both WEs and counter electrodes (CEs) due to their high corrosion resistance and electrical conductivity for medical applications,^46–48^ while quasi Ag/AgCl wires can be used as reference electrodes (REs), as in our earlier study.^40^

For initial simulations, the WE and CE were set to have the same dimensions, with diameters of 0.245 mm and a length of 32.6 mm. The WE was placed in the center of the hub, and the CE 1.47 mm away from the center of the hub. Due to distinct dimensions of each compartment, the assembly did not exhibit an axially symmetric geometry (see Fig. 1B); therefore a fully three-dimensional (3D) geometry was employed. Although the constructed 3D model accounts for concentration distribution across all dimensions, the most important gradient is over the e-catheter length. Electrode and e-catheter dimensions were selected based on a prototype developed in our laboratory. For modeling, the solution within the catheter is assumed to be stagnant.

Electrochemical generation of HOCl, its dissociation and degradation reactions, and other reactions that take place in the e-catheter, and the flow of the electrons, are represented in Fig. 1C. In an electrochemical cell, electrons flow from the anode to the cathode, or from the WE to the CE in the e-catheter system. The anode acts as an electron sink collecting electrons released by oxidation reactions. These electrons are transferred through an external circuit to the cathode. The cathode acts as an electron donor for reduction reactions requiring electrons. Chemical species generated by electrochemical reactions may dissociate and degrade due to thermodynamic instability.

Interactions with biological agents such as bacterial cells, biofilm components, and human blood components, or chemical agents that might be co-present in catheters, which could impact HOCl generation and degradation, were not included in the modeling.

### Model reactions

#### Electrochemical reactions

The e-catheter is filled with 0.9% NaCl, which dissociates in water into Na^+^ and Cl^−^ ions (154.04 mol m^−3^). Oxidation of Cl^−^ into HOCl occurs on the WE,^49^ Eq. 1. Since the generated HOCl concentration does not exceed the solubility of Cl_2_ gas (60–90 mol m^−3^),^50^ Cl_2_ gas does not form.^45^

\begin{eqnarray*}{{\mathrm{Cl}}}^{-}+{{\mathrm{H}}}_{2}{\mathrm{O}}\rightleftarrows {\mathrm{HOCl}}+{{\mathrm{H}}}^{+}+2{{\mathrm{e}}}^{-}\,{i}_{we}\end{eqnarray*}

The hydrogen evolution reaction (HER) occurs on the CE,^45^ Eq. 2:

\begin{eqnarray*}2{{\mathrm{H}}}^{+}+2{{\mathrm{e}}}^{-}\rightleftarrows {{\mathrm{H}}}_{2}\,{i}_{ce}\end{eqnarray*}

Butler-Volmer expressions were applied for current densities *i*_*we*_ and *i*_*ce*_, as shown in Eq. 3 (WE, *we*) and Eq. 4 (CE, *ce*), in the respective electrode domains, with *i*_*0,we*_ and *i*_*0,ce*_, the exchange current densities, and *α*_*we*_ and *α*_*ce*_, the transfer coefficients:

\begin{eqnarray*}{i}_{we}={i}_{0,we}\left(\exp \left(\displaystyle \frac{\left(1-{\alpha }_{we}\right)F}{RT}{\eta }_{we}\right)-\exp \left(\displaystyle \frac{-{\alpha }_{we}F}{RT}{\eta }_{we}\right)\right)\end{eqnarray*}

\begin{eqnarray*}{i}_{ce}={i}_{0,ce}\left(\exp \left(\displaystyle \frac{\left(1-{\alpha }_{ce}\right)F}{RT}{\eta }_{ce}\right)-\exp \left(\displaystyle \frac{-{\alpha }_{ce}F}{RT}{\eta }_{ce}\right)\right)\end{eqnarray*}

*η*_*we*_ and *η*_*ce*_ are the respective overpotentials, defined as, Eqs. 5 and 6:

\begin{eqnarray*}{\eta }_{we}={\phi }_{we}-\phi -{E}_{we}\end{eqnarray*}

\begin{eqnarray*}{\eta }_{ce}={\phi }_{ce}-\phi -{E}_{ce}\end{eqnarray*}

Electrode potentials, *ϕ*_*we*_ and *ϕ*_*ce*,_ were set constant due to high conductivity of the possible electrode materials, titanium (2.38 × 10^6^ S m^−1^) and gold (4.10 × 10^7^ S m^−1^).^47^ Due to high conductivity of the saline solution,^51^ aqueous electrolyte potential gradients are negligible, thus $\phi =0$ in the whole model. Redox potentials, *E*_*we*_ and *E*_*ce*_, were computed according to the Nernst equation, from standard redox potentials, ${E}_{{we}}^{0}$ and ${E}_{{ce}}^{0},$ and local concentrations of the chemical species participating in the electrochemical reaction, Eqs. 7 and 8:

\begin{eqnarray*}{E}_{we}={E}_{we}^{0}-\displaystyle \frac{RT}{2F}\,\mathrm{ln}\left[{\left(\displaystyle \frac{{c}_{{H}^{+}}}{{c}_{{H}^{+},ref}}\right)}^{-1}{\left(\displaystyle \frac{{c}_{HOCl}}{{c}_{HOCl,ref}}\right)}^{-1}\left(\displaystyle \frac{{c}_{C{l}^{-}}}{{c}_{C{l}^{-},ref}}\right)\right]\end{eqnarray*}

\begin{eqnarray*}{E}_{ce}={E}_{ce}^{0}-\displaystyle \frac{RT}{2F}\,\mathrm{ln}\left[{\left(\displaystyle \frac{{c}_{{H}^{+}}}{{c}_{{H}^{+},ref}}\right)}^{-2}\left(\displaystyle \frac{{c}_{{H}_{2}}}{{c}_{{H}_{2},ref}}\right)\right]\end{eqnarray*}

From current densities, local volumetric rates of electrochemical reactions were calculated in the electrode domains according to Faraday’s law, Eq. 9:

\begin{eqnarray*}{r}_{e,we}=-\displaystyle \frac{{i}_{we}}{2F},{r}_{e,ce}=-\displaystyle \frac{{i}_{ce}}{2F}\end{eqnarray*}

From these reaction rates, rates for individual chemical species, ${r}_{e,i},$ were calculated according to the reaction stoichiometry.

#### Chemical reactions

Two acid-base equilibria were considered in the computational domain for pH calculation and chemical speciation, Eqs. 10 and 11:

\begin{eqnarray*}{\mathrm{HOCl}}\rightleftarrows {{\mathrm{ClO}}}^{-}+{{\mathrm{H}}}^{+}\,{r}_{a,HOCl}={k}_{a}\left({c}_{HOCl}-\displaystyle \frac{{c}_{Cl{O}^{-}}{c}_{{H}^{+}}}{{K}_{a,HOCl}}\right)\end{eqnarray*}

\begin{eqnarray*}{{\mathrm{H}}}_{2}{\mathrm{O}}\rightleftarrows {{\mathrm{HO}}}^{-}+{{\mathrm{H}}}^{+}\,{r}_{a,{H}_{2}O}={k}_{a}^{* }\left(1-\displaystyle \frac{{c}_{H{O}^{-}}{c}_{{H}^{+}}}{{K}_{a,{H}_{2}O}}\right)\end{eqnarray*}

Reactions were treated kinetically, with finite but large reversible rates (by setting an arbitrarily large value of the rate constants, ${k}_{a}$ and ${k}_{a}^{* }$), reaching a close-to-equilibrium state at all times and locations according to their equilibrium constants, *K*_*a*_. Degradation of HOCl was proposed to occur as follows, Eqs. 12–15.^52,53^

\begin{eqnarray*}\begin{array}{cc}\mathrm{HOCl}\to {{\mathrm{H}}}^{+}+{\mathrm{Cl}}^{-}+{\frac{1}{2}{\mathrm{O}}}_{2} &amp; \,{r}_{d,{K}_{0}}={K}_{0}{c}_{{HOCl}}\end{array}\end{eqnarray*}

\begin{eqnarray*}\begin{array}{cc}2\mathrm{HOCl}\to 2{{\mathrm{H}}}^{+}+{\mathrm{Cl}}^{-}+{{\mathrm{ClO}}_{2}}^{-} &amp; \,{r}_{d,{K}_{1}}={K}_{1}{{c}_{{HOCl}}}^{2}\end{array}\end{eqnarray*}

\begin{eqnarray*}\begin{array}{cc}\mathrm{HOCl}+{\mathrm{ClO}}_{2}^{-}\to \mathrm{C}{\mathrm{l}}^{-}+{{\mathrm{H}}}^{+}+{{\mathrm{ClO}}_{3}}^{-} &amp; \,{r}_{d,{K}_{2}}={K}_{2}{c}_{{HOCl}}{c}_{{{ClO}}_{2}^{-}}\end{array}\end{eqnarray*}

\begin{eqnarray*}\begin{array}{cc}\mathrm{HOCl}+\mathrm{C}{\mathrm{lO}}^{-}\to {{\mathrm{H}}}^{+}+{\mathrm{Cl}}^{-}+{\mathrm{ClO}}_{2}^{-} &amp; \,{r}_{d,{K}_{3}}={K}_{3}{c}_{{HOCl}}{c}_{{{ClO}}^{-}}\end{array}\end{eqnarray*}

HCl, HClO_2_ and HClO_3_ are strong acids, considered in the model as completely dissociated into H^+^, Cl^−^, ClO_2_^−^ and ClO_3_.

### Model balances

In the aqueous e-catheter domain, transport of chemical species takes place by diffusion only, accompanied by specific chemical and electrochemical reactions. The following chemical species were included: HOCl, ClO^−^, ClO_2_^−^, ClO_3_^2−^, O_2_, H^+^, H_2_, HO^−^, Cl^−^, and Na^+^.^54^ The saline solution was left unbuffered so that generation of H^+^ resulting from Cl^−^ oxidation and from HOCl degradation reactions would affect local pH. Time-dependent material balances were set for each chemical component, *i*, including diffusion, together with the net reaction rates, *R*_*i*_, Eq. 16.

\begin{eqnarray*}\displaystyle \frac{\partial {c}_{i}}{\partial t}={D}_{i}{{\mathrm{\nabla }}}^{2}{c}_{i}+{R}_{i}\end{eqnarray*}

*c*_*i*_ is the molar concentration, and *D*_*i*_ is the diffusion coefficient. Diffusion coefficients in the hub were considered equal to those of respective species in water at 25 °C. As the catheter will be ultimately inserted into humans, temperature of the hub and connector was set to 25 °C, temperature of the tube was set to 37 °C, and temperature effect on the diffusion was included (with T in K), Eq. 17:^55^

\begin{eqnarray*}{D}_{i}={D}_{i,298K}\times {\left(\displaystyle \frac{T}{298K}\right)}^{3/2}\end{eqnarray*}

Net reaction rates for each component, *R*_*i*_, are formed by stoichiometric summation of volumetric chemical rates participating in the respective domain, Eq. 18:

\begin{eqnarray*}{R}_{i}=\displaystyle \displaystyle \sum _{ja}{\nu }_{ja,i}{r}_{a,i}+\displaystyle \displaystyle \sum _{jd}{\nu }_{jd,i}{r}_{d,i}\end{eqnarray*}

with *ν*_*ja,i*_ and *ν*_*jd,i*_, the stoichiometric coefficients, in reactions (10) to (15).

No-flux conditions (${\boldsymbol{n}}\bullet {{\boldsymbol{J}}}_{i}=0$) were set for all species on all model walls, except on electrode surfaces, where the diffusive flux, ${{\boldsymbol{J}}}_{i}=-{D}_{i}{\mathrm{\nabla }}{c}_{i},$ equals the electrode reaction rate for component *i* ($-{\boldsymbol{n}}\bullet {{\boldsymbol{J}}}_{i}={r}_{e,i}$).

Initial concentrations of H^+^, OH^−^, Cl^−^, and Na^+^ were calculated from initial composition of the electrolyte solution used (pH = 5.5, NaCl 154.04 mM), while other species concentrations were set to zero.

### Model meshing

The model mesh was constructed to balance computational efficiency with a sufficiently accurate representation. The top surface of the e-catheter hub was covered by a free triangular mesh swept along the e-catheter: the body of the hub with 30 elements, the bottom of the hub (titanium wire-free) with 2 elements, the top of the Luer lock (connector) with 5 elements, the bottom of the Luer lock with 10 elements, and the tube with 200 elements.

### Model strategy and parameters

COMSOL Multiphysics was used as the simulation platform. Model parameters were selected based our previous work and that of others, as listed in Table I. Electrochemical parameters can be experimentally obtained from a Tafel diagram, which assumes “that the concentrations at the electrode are practically equal to the concentrations in the bulk electrolyte.”^60^ Given that this study aims to highlight parameters that would affect HOCl generation in the e-catheter, experimental data was not used to estimate the reaction rates. For comparison of the concentration results, local concentration gradients over the e-catheter length, volume average concentrations, and 3D concentration gradients were considered. For volume average concentration calculations, the concentration of interest was divided by the total volume of the mixture for different parts of the e-catheter using COMSOL Multiphysics®.

**Table I.** Parameters used in the HOCl generating e-catheter model.

Parameter	Description	Value	Units	Source
Geometry				

${d}_{{we}}$	WE wire thickness	2.45 × 10^−4^	m	This work
${d}_{{ce}}$	CE wire thickness	2.45 × 10^−4^	m	This work
${d}_{{hub}}$	Hub diameter	5.4 × 10^−3^	m	This work
${d}_{{luer},{top}}$	Connector top part diameter	4.0 × 10^−3^	m	This work
${d}_{{luer},{bottom}}$	Connector bottom part diameter	1.6 × 10^−3^	m	This work
${d}_{{tube}}$	Tube diameter	1.6 × 10^−3^	m	This work
${L}_{{we}}$	WE wire length	3.2 × 10^−2^	m	This work
${L}_{{ce}}$	CE wire length	3.2 × 10^−2^	m	This work
${L}_{{hub}}$	Hub length	3.26 × 10^−2^	m	This work
${L}_{{luer},{top}}$	Connector top part length	6.4 × 10^−3^	m	This work
${L}_{{luer},{bottom}}$	Connector bottom part length	1.22 × 10^−2^	m	This work
${h}_{{tube}}$	Tube length	0.3	m	This work

Reactions				

${k}_{{HOCl}}$	Rate constant	3.4	m^3^/mol/s	25 °C^56^
${k}_{{ClO}}$	Rate constant	44000	m^3^/mol/s	25 °C^56^
${E}_{a,{HOCl}}$	Activation energy	7.7	kcal	56
${E}_{a,{{ClO}}^{-}}$	Activation energy	11.6	kcal	56
${k}_{a}$	Rate constant	1000	s^−1^	large value
${k}_{{a}* }$	Rate constant	1000	mol/m^3^.s	large value
${K}_{a,{H}_{2}O}$	Equilibrium constant	1 × 10^−8^	mol^2^/m^6^	
${K}_{a,{HOCl}}$	Equilibrium constant	3.16 × 10^−5^	mol/m^3^	57
${K}_{0}$	Rate constant	4.65 × 10^−4^	1/min	30 °C^52^
${K}_{1}$	Rate constant	4.5 × 10^−1^	L/mol/min	30 °C^52^
${K}_{2}$	Rate constant	3 × 10^−3^	L/mol/min	30 °C^52^
${K}_{3}$	Rate constant	7.5 × 10^−6^	L/mol/min	60 °C^53^
${E}_{a,{K}_{0}}$	Activation energy	19	kcal	52
${E}_{a,{K}_{1}}$	Activation energy	15	kcal	52
${E}_{a,{K}_{2}}$	Activation energy	15	kcal	52
${E}_{a,{K}_{3}}$	Activation energy	15	kcal	53

Electrochemical				

${\alpha }_{{we}}$	Charge transfer coefficient, WE reaction	0.95	—	Chosen^48^
${\alpha }_{{ce}}$	Charge transfer coefficient, CE reaction	0.05	—	Chosen^48^
${E}_{{we}}^{0}$	Standard reduction potential, WE reaction $\mathrm{HOCl}+{{\mathrm{H}}}^{+}+2{\mathrm{e}}^{-}\rightleftarrows \mathrm{C}{\mathrm{l}}^{-}+{{\mathrm{H}}}_{2}{\mathrm{O}}$	1.297	V_Ag/AgCl_	54
${E}_{{ce}}^{0}$	Standard reduction potential, CE reaction $2{\mathrm{H}}^{+}+2{\mathrm{e}}^{-}\rightleftarrows {{\mathrm{H}}}_{2}$	−0.197	V_Ag/AgCl_	54
${i}_{0,{we}}$	Exchange current density, WE reaction	0.032	A/m^2^	Chosen^48^
${i}_{0,{ce}}$	Exchange current density, CE reaction	0.003	A/m^2^	Chosen^48^

Electrical				

${V}_{a,{we}}$	WE potential	1.5	V_Ag/AgCl_	58
${V}_{a,{ce}}$	CE potential	−0.6	V_Ag/AgCl_	58

Concentrations^a)^				

${{pH}}_{0}$	Initial pH	5.5	—	This work
${C}_{0,{NaCl}}$	Initial NaCl concentration	154.04	mol/m^3^	This work

Diffusion				

${D}_{{{OH}}^{-}}$	OH^−^ diffusion coefficient	5.27 × 10^−9^	m^2^/s	25 °C^54^
${D}_{{H}^{+}}$	H^+^ diffusion coefficient	9.31 × 10^−9^	m^2^/s	25 °C^54^
${D}_{{{Na}}^{+}}$	Na^+^ diffusion coefficient	1.334 × 10^−9^	m^2^/s	25 °C^54^
${D}_{{{Cl}}^{-}}$	Cl^−^ diffusion coefficient	1.032 × 10^−9^	m^2^/s	25 °C^54^
${D}_{{{ClO}}^{-}}$	ClO^−^ diffusion coefficient	1 × 10^−9^	m^2^/s	—
${D}_{{HOCl}}$	HOCl diffusion coefficient	1.14 × 10^−9^	m^2^/s	25 °C^59^
${D}_{{H}_{2}}$	H_2_ diffusion coefficient	6.30 × 10^−9^	m^2^/s	25 °C^55^
${D}_{{{ClO}}_{2}^{-}}$	ClO_2_^−^ diffusion coefficient	1 × 10^−9^	m^2^/s	—
${D}_{{{ClO}}_{3}^{-}}$	ClO_3_^−^ diffusion coefficient	1 × 10^−9^	m^2^/s	—
${D}_{{AB},T}$	${D}_{{AB},T}={D}_{{AB},298K}x({\frac{T}{298K})}^{3/2}$	${D}_{{AB}}\propto {T}^{3/2}$	m^2^/s	55

a) Initial concentrations of all ions were computed by solving the system of mole balances, charge balance, and mass action laws using ${{pH}}_{0}$ and ${C}_{0,{NaCl}}.$

## Results and Discussion

Electrochemical generation of HOCl inside an e-catheter was simulated using the EHAP model, implemented in COMSOL Multiphysics®. The model was used to define variables for e-catheter operation in addition to predicting HOCl concentrations, namely polarization time, diffusion of HOCl, operational voltage, WE length, and WE surface area.

### HOCl concentration profiles in the e-catheter

**Figure 2.**
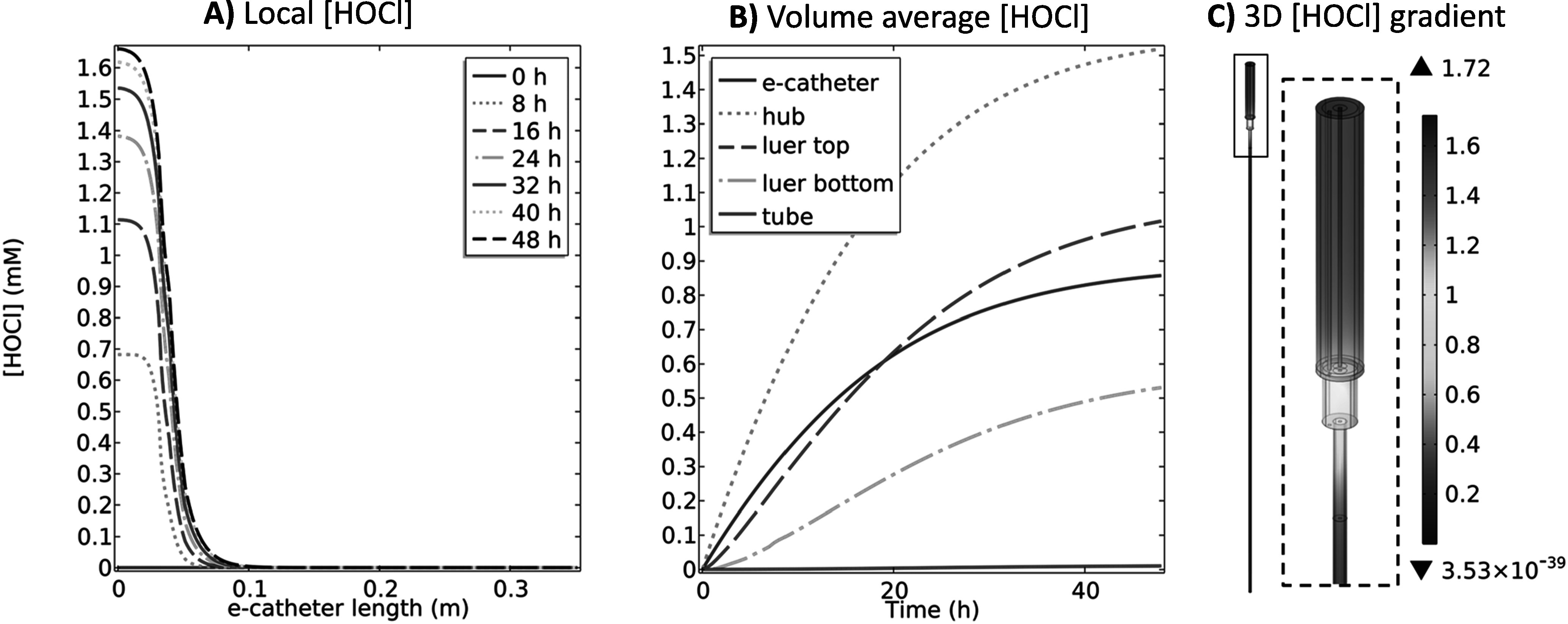
(A) Local HOCl (mM) profiles at 0, 8, 16, 24, 32, 40 and 48 h, (B) volume average HOCl (mM) profiles over 48 h and (C) 3D HOCl (mM) gradient at 48 h in the e-catheter, at 1.5 V_Ag/AgCl_ (T_hub_ = 25 °C and T_tube_ = 37 °C) (d_hub,WE_ = 2.45 × 10^–4 ^m, L_hub,WE_ = 3.26 × 10^–2^ m, d_tube,WE_ = 0 mm, and L_tube,WE_ = 0 mm).

The first approach to investigate HOCl concentration distribution within the e-catheter system was modeled by locating the WE and CE in the catheter hub. This approach was selected to prevent intraluminal infections. Intraluminal infections start from the hub and occur when the catheter hub is not handled properly, typically within one week after catheter placement.^61^ Therefore, preventing bacterial or fungal colonization in the hub, and subsequent dissemination to the other catheter compartments, may prevent spread of infection. The HOCl concentration profile throughout the e-catheter was monitored (Fig. 2) under constant potential (1.5 V_Ag/AgCl_) conditions for 48 h.^40^

Figure 2A shows the local HOCl concentration profiles in the e-catheter system at 0, 8, 16, 24, 32, 40 and 48 h. Zero length refers to the top of the e-catheter, and 0.35 m to the bottom of the tube (Fig. 1). HOCl concentration profiles showed variation between different parts of the e-catheter. The concentration was highest at the top of the catheter and gradually increased with escalating polarization time at 1.5 V_Ag/AgCl_; its accumulation rate ($\partial C/\partial t$) decreased with increased time (Fig. 2A). HOCl concentrations at any given time were averaged for the entire e-catheter—hub, Luer lock top and bottom (connectors), and tube—and presented as the volume average HOCl concentration in Fig. 2B. The highest HOCl concentration was observed in the hub due to the location of the WE. The volume average HOCl concentration in the e-catheter was lower than the hub concentration due to the low concentration profile in the other parts. Figure 2C shows the 3D HOCl concentration gradient as a color map at 48 h for the entire e-catheter, including the hub, connectors, and tube. After 48 h of constant polarization, the highest concentration (1.72 mM) was observed near the WE surface in the hub, while the lowest concentration (∼0 mM) was observed at the bottom of the tube (Fig. 2C) at 48 h. Although Fig. 2B shows that the volume average HOCl concentration in the tube increased over time, Figs. 2A and 2C show that the HOCl concentration at 48 h was higher for the upper part of the tube, and decreased with increasing tube length, reflecting the limited diffusion of HOCl for 48 h.

In previous studies, HOCl (0.03 mM–3.97 mM) showed activity against different types of pathogens, including bacteria and fungi.^30,34,62–64^ In one study, the minimum inhibitory concentration (MIC), minimum biofilm inhibitory concentration (MBIC), and minimum biofilm bactericidal concentration (MBBC) ranged from 0.5 to 1.99 mM, 0.66 to ≥3.97 mM and 0.66 to ≥3.97 mM respectively for 27 bacterial isolates [∼5  ×  10^5^ colony forming units (CFU) of bacteria] ml^−1^ over 18–24 h at 37 °C.^34^ In another study, the minimum bactericidal concentrations (MBC) ranged from 1.9 to 53.3 μM for a broad spectrum of 20 bacterial and fungal microorganisms (5 × 10^5^ CFU ml^−1^), in 1 hour at 25 °C.^30^ Similarly, another study showed that HOCl is effective against planktonically growing microorganisms, including 13 bacterial, 3 fungal and 3 yeast species (∼10^8^ CFU ml^−1^) at 0.03, 0.24 and 1.43 mM, respectively after 300 seconds of exposure.^62^ Results in Fig. 2 demonstrated that the designed e-catheter is expected to prevent infections in the hub, based on predicted concentration generation.

Additionally, local concentration profiles of O_2_, ClO_2_^−^, H_2_ and pH were monitored (Fig. S.1A) due to degradation of HOCl, according to Eqs. 12–15, and the hydrogen evolution reaction, according to Eq. 2. The local concentrations of O_2_, ClO_2_^−^ and H_2_ were higher in the hub compared to the connector and tube at 48 h. ClO_2_^−^ is mainly produced by HOCl degradation, according to Eq. 13, instead of the HOCl and ClO^−^ reaction, given as Eq. 15, due to the lack of ClO^−^ (Figs. S.2G–S.2I). Similar to the HOCl concentration profiles, ClO_2_^−^ concentration was highest (1.27 mM) near the WE surface in the hub and lowest in the tube (∼0 mM) (Figs. S.2A–S.2C) at 48 h, and its concentration increased with increasing HOCl concentration (Fig. S.1A). The effective activity of ClO_2_^−^ (chlorine dioxide), also known as ClO_2_ or HClO_2,_ against pathogens was shown in previous studies.^65–67^ The notation ClO_2_^−^ is used for chlorine dioxide in the rest of the article for simplification. In one study, 0.015 and 0.02 mM ClO_2_^−^ were effective against 19 different microbial species in planktonic and biofilm form, respectively, after 18 h of treatment,^67^ suggesting that HOCl and ClO_2_^−^ concentrations generated in the hub would be effective for infection prevention as well as biofilm eradication.

pH varies over time and across e-catheter locations due to diffusion and H^+^ dynamics. Modeling via COMSOL offers insights into pH changes that are challenging to measure experimentally due to limitations in physical access. pH decreased over time in all compartments due to HOCl generation from Cl^−^ oxidation, according to Eq. 1, in the hub (Figs. S.2D&S.2E), its dissociation, as given in Eq. 10, and degradation, as given in Eqs. 12–15. The decrease was less significant in the tube compared to the other compartments due to local generation in the hub and limited diffusion. The highest and lowest pH were 4.98 and 2.33 in the tube and hub, respectively (Fig. S.2F) at 48 h. Lower pH indicates higher concentrations of H^+^, which is generated when Cl^−^ is oxidized to HOCl and H^+^. HOCl is more stable in acidic environments in HOCl/ClO^−^ equilibria.^45,68^ These differences in pH might affect how HOCl is distributed and its effectiveness in the catheter. Therefore, ClO^−^ concentration, which is in equilibrium with HOCl, was also monitored. Figures S.2G shows the local ClO^−^ concentration profile throughout the e-catheter. The results show that the ClO^−^ concentration was close to zero in all compartments due to a low pH. Although pH (Fig. S.2F) is relatively higher in the tube, the negligible ClO^−^ concentrations demonstrate that HOCl generated in the hub needs a longer time to diffuse in the tube.

The data in Figs. 2 and S.2 suggest that, while HOCl generation was efficient in the catheter hub, where the electrode was located, there was limited diffusion into the tube during the polarization period. Accordingly, this configuration can be considered for the prevention of hub-originating intraluminal infections.

### Factors influencing HOCl production and distribution in the e-catheter

The effect of waiting for HOCl to diffuse, changing the operating voltage, and changing the WE length and thickness on HOCl generation and distribution within the e-catheter was investigated with the EHAP model.

#### Diffusion of HOCl post-polarization

Forty-eight hours of polarization at 1.5 V_Ag/AgCl_ (Fig. 2) followed by no polarization was selected to investigate the diffusion of HOCl in the e-catheter for 120 h.

**Figure 3.**
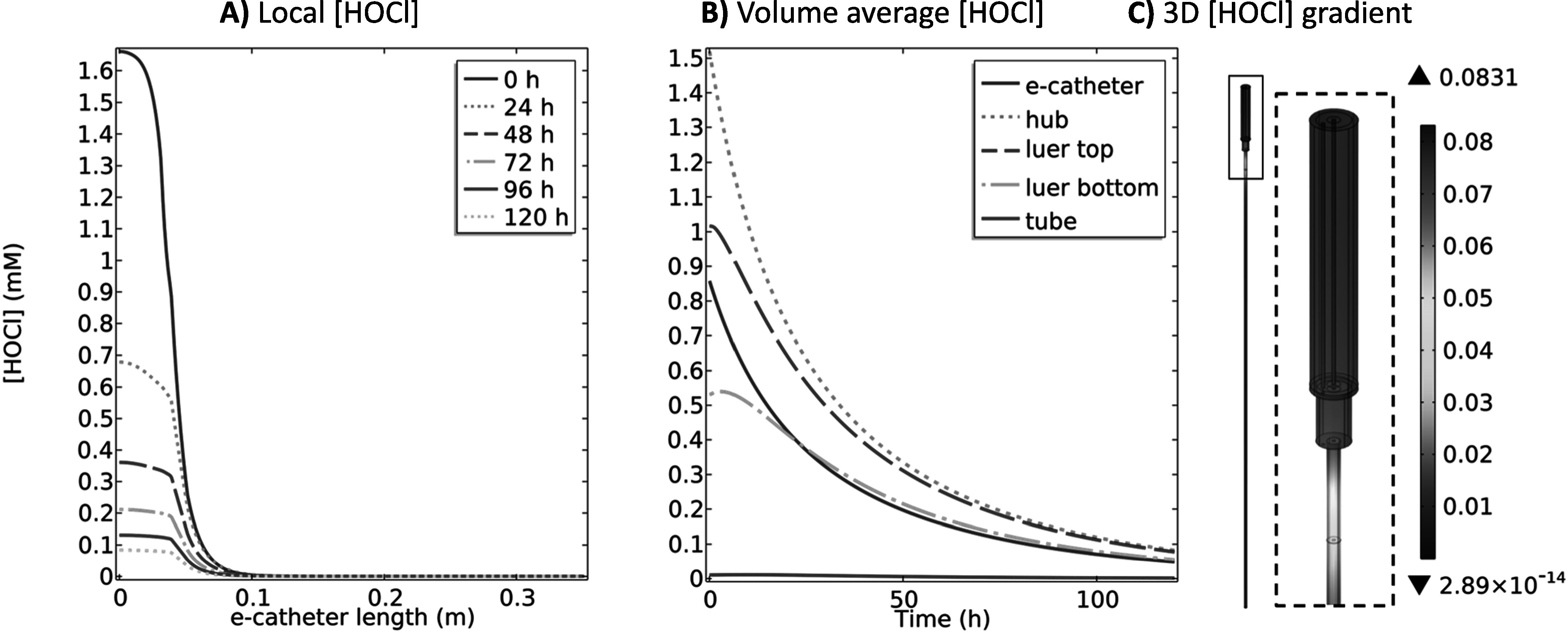
(A) local HOCl (mM) profiles at 0, 24, 48, 72, 96 and 120 h (no polarization), (B) volume average HOCl (mM) profiles over 120 h at different compartments of the e-catheter after 48 h polarization at 1.5 V_Ag/AgCl_, and (C) 3D HOCl (mM) gradient at 120 h in the e-catheter, (T_hub_ = 25 °C and T_tube_ = 37 °C) (d_hub,WE_ = 2.45 × 10^–4 ^m, L_hub,WE_ = 3.26 × 10^–2^ m, d_tube,WE_ = 0 mm and L_tube,WE_ = 0 mm).

Figure 3 shows HOCl concentration profiles in the e-catheter during 120 h with no polarization. The local HOCl concentration profile in the hub decreased over time (Fig. 3A). Similarly, the volume average HOCl concentrations decreased in all compartments over time (Fig. 3B). This decrease was due to uncatalyzed degradation of HOCl according to Eqs. 12–15. At 120 h, maximum HOCl concentrations in the hub and tube were ∼0.08 and ∼0 mM, respectively (Fig. 3C). Figure S.1B shows O_2_ and ClO_2_^−^ concentrations increased throughout the e-catheter compared to the previous observation (Fig. S.1A) due to degradation of HOCl according to the Eqs. 12 and 13, and 15, respectively, and diffusion of the species. However, O_2_ and ClO_2_^−^ concentrations remained as in the previous observation (Fig. 2) at the bottom of the catheter tube. The model showed that both O_2_ and ClO_2_^−^ concentrations increased in the hub. ClO_2_^−^ is generated via two pathways of HOCl degradation: (1) direct HOCl decomposition, and (2) reaction of HOCl with ClO^−^ according to Eqs. 13 and 15, respectively. Therefore, ClO_2_^−^ concentration increased while HOCl and ClO^−^ concentrations decreased (Figs. S.3A & S.3B, 3A & 3B and S.3G & S.3H). ClO^−^ concentration was monitored as close to zero in all compartments (Fig. S.3I), therefore ClO_2_^−^ generation was mainly due to direct HOCl decomposition as in Eq. 13. The model showed that the ClO_2_^−^ concentration is higher in the hub compared to the tube and is ∼0 mM at the bottom of the tube (Figs. S.3A–S.3C). ClO_2_^−^ should not pose any risks to human health for the selected operational conditions since it is not present at the bloodstream interface. pH decreased promptly in the tube and increased slightly in the other compartments over 120 h (Figs. S.3D & S.3E); the highest and lowest pH were observed as 3.9 and 2.39 in the tube and hub, respectively (Fig. S.3F) at 120 h. Decreasing pH in the tube from 4.98 (Fig. S.1A) to 3.9 (Fig. S.1B) over time implies diffusion of H^+^ from the hub to the tube. Although pH changed in the tube, the ClO_2_^−^ concentration remained ∼0 mM at the bottom of the tube (Fig. S.3C) at 120 h, showing limited diffusion of larger molecules compared to H^+^. Low HOCl and ClO_2_^−^ concentrations (∼0 mM) at the bottom of the tube are practically desired to prevent toxic effects at the bloodstream connection site. Increasing WE potential or inserting a WE into the tube is a potential alternative approach to increase HOCl concentration inside the tube.

#### Working electrode potential

**Figure 4.**
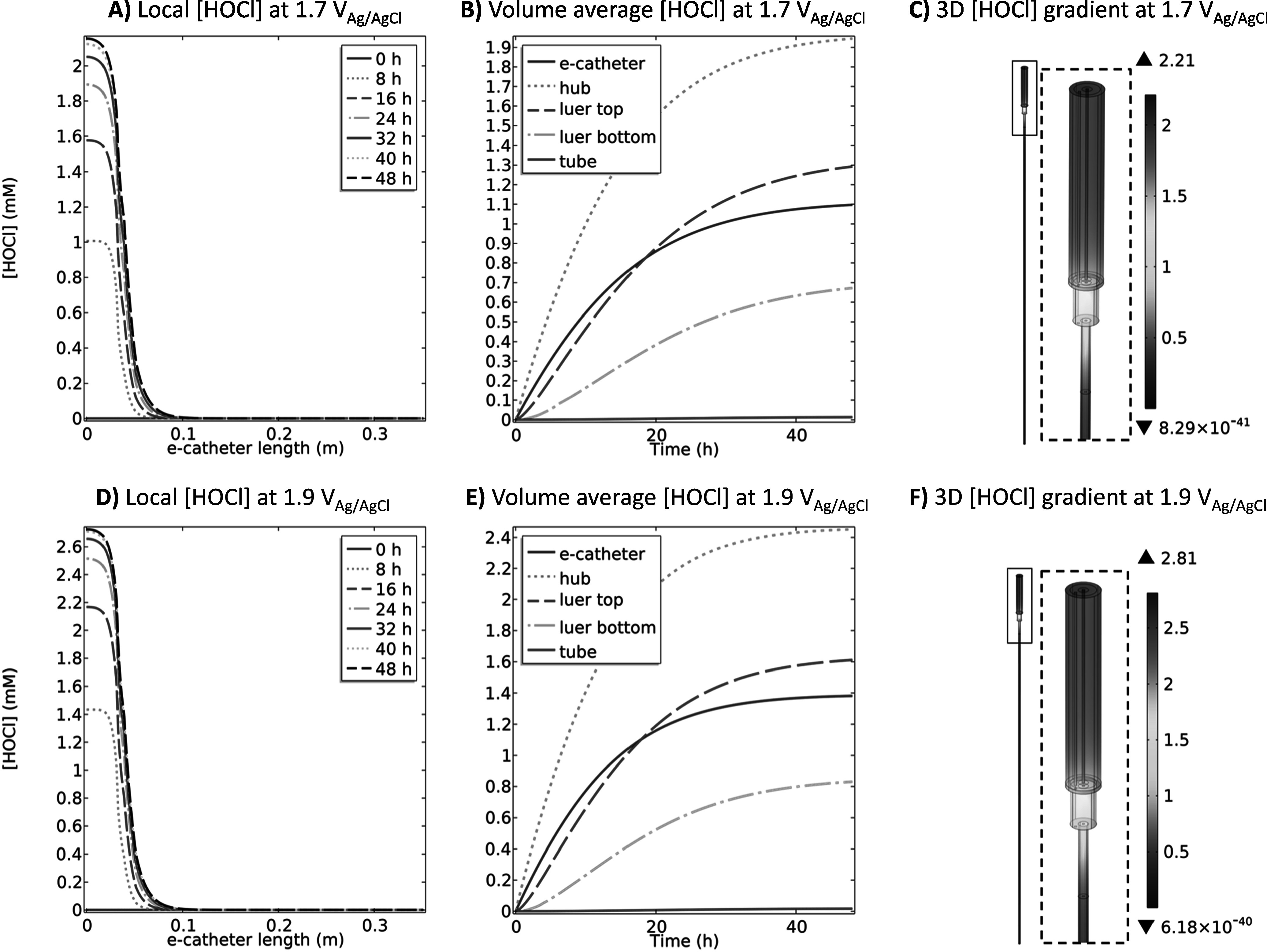
(A) local HOCl (mM) at 1.7 V_Ag/AgCl_ and D) local HOCl (mM) at 1.9 V_Ag/AgCl_ at 0, 8, 16, 24, 32, 40 and 48 h, (B) volume average HOCl (mM) at 1.7 V_Ag/AgCl_ and (E) volume average HOCl (mM) at 1.9 V_Ag/AgCl_ profiles over 48 h at different compartments of the e-catheter and (C) 3D HOCl (mM) gradient at 1.7 V_Ag/AgCl_ and (F) 3D HOCl (mM) gradient at 1.9 V_Ag/AgCl_ at 48 h in the e-catheter (T_hub_ = 25 °C and T_tube_ = 37 °C) (d_hub,WE_ = 2.45 × 10^–4 ^m, L_hub,WE_ = 3.26 × 10^–2^ m, d_tube,WE_ = 0 mm and L_tube,WE_ = 0 mm).

The overpotential (*η*_*we*_ and *η*_*ce*_) in an electrochemical system is the driving force behind either reduction or oxidation reactions.^69^ The difference between the formal potential for a reaction and the potential applied between the WE and RE is referred to as the overpotential.^70^ Applying more positive potentials relative to the system-specific onset potential causes HOCl production rate to increase until diffusion limitations occur according to the previously published data and Eq. 3.^69^ The literature shows that HOCl production begins at the onset potential of 1.1 V_Ag/AgCl_ on a platinum wire in a saline solution.^40^ As WE potential becomes more positive, the reaction rate increases.^40^ In order the elevate the transport phenomena and deliver HOCl to the upper parts of the tube, the HOCl concentration in the hub should be higher. This means that more driving force (*η*_*we*_) should be applied to the WE to increase HOCl generation rate. Therefore, in addition to 1.5 V_Ag/AgCl_ (Fig. 2), applying 1.7 V_Ag/AgCl_ and 1.9 V_Ag/AgCl_ to the WE and its effects on the HOCl concentration profiles over 48 h was simulated (Fig. 4).

Figures 4A & 4D show local HOCl concentration profiles for 1.7 V_Ag/AgCl_ and 1.9 V_Ag/AgCl_, respectively. Predicted local HOCl concentration in the hub increased with increasing potential applied. Volume average concentrations were also increased in all compartments over time with increasing applied potential except for in the tube (Figs. 4B & 4E). Figures 4C & 4F show that HOCl concentrations reach a maximum of 2.21 and 2.81 mM for 1.7 V_Ag/AgCl_ and 1.9 V_Ag/AgCl_, respectively in the hub, but remain at ∼0 mM in the tube at 48 h. Similarly, O_2_ and ClO_2_^−^ concentrations increased in the hub while pH decreased with increasing the applied potential (Figs. S.1C & S.1D) compared to 1.5 V_Ag/AgCl_ (Fig. S.1A). Although the O_2_ and ClO_2_^−^ concentrations in the hub increased significantly for both potentials, their concentration in the bottom of the tube remained at ∼0 mM (Figs. S.1C & S.1D and S.4A–S.4F). The pH ranged between 4.2 and 4 in the tube part for 1.7 V_Ag/AgCl_ (Figs. S.4J & S.4H) and 1.9 V_Ag/AgCl_ (Figs. S.4J & S.4K), respectively at 48 h. This indicates that the transport of H^+^ was elevated by promoting Cl^−^ oxidation, according to Eq. 1, with a higher driving force (*η*_*we*_ or overpotential). However, low concentrations (∼0 mM) of HOCl and ClO_2−_ in the bottom of the tube indicate diffusion limitation of larger molecules. Therefore, even though the transport of species enhanced with an increase in the concentration, applying higher potentials generate low HOCl and ClO_2_^−^ concentrations at the bottom of the tube. Thus, the selected operating conditions should prevent pathogens from entering and forming biofilms without reaching hematotoxic levels at the bottom of the tube.

#### Length of the working electrode

To increase HOCl concentrations in the tube, the WE can be inserted inside the tube. By doing so, HOCl delivery to the tube will not depend on diffusion. To simulate maximum HOCl generation in the tube, a WE length of 0.33 m was simulated, which is equivalent to the length of the tube with the hub attached (Fig. 1).

**Figure 5.**
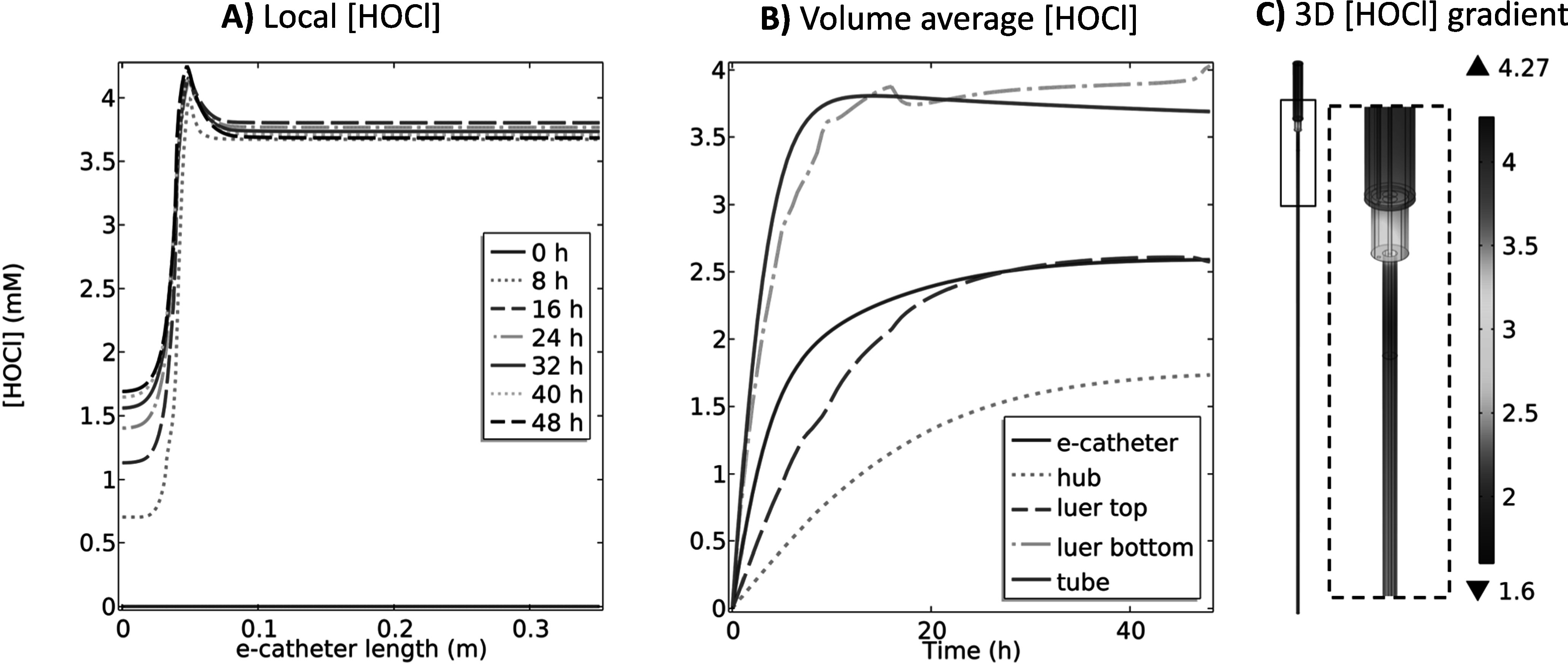
(A) local HOCl (mM) profiles at 0, 8, 16, 24, 32, 40 and 48 h, (B) volume average HOCl (mM) profiles over 48 h and (C) 3D HOCl (mM) gradient at 48 h in the e-catheter, at 1.5 V_Ag/AgCl_ (T_hub_ = 25 °C and T_tube_ = 37 °C) (T_hub_ = 25 °C and T_tube_ = 37 °C) (d_hub,WE_ = 2.45 × 10^–4 ^m, L_hub,WE_ = 3.26 × 10^–2^ m, d_tube,WE_ = 2.45 × 10^–4 ^m and L_tube,WE_ = 0.3 m).

Figure 5 illustrates a significant increase in HOCl concentration observed in the tube while the HOCl concentration in the hub remained similar to the previous observation (Fig. 2). This activity is mainly related to the extension of WE length. The local HOCl concentration profiles show that the HOCl concentration peaked in the connector (Luer lock bottom) and was higher in the tube compared to the hub (Fig. 5A). This was due to the higher ratio between the WE surface area and the volume of the NaCl solution in the connector, compared to other parts. The volume average HOCl concentration in the tube increased for the first 17 h and reached ∼3.7 mM, then slowly decreased over time (Fig. 5B), and the highest and lowest HOCl concentrations in the e-catheter were observed as 4.27 and 1.6 mM, respectively (Figs. 5A & 5C). The model showed that O_2_ and ClO_2_^−^ concentrations markedly increased in the tube due to the high electrode area to solution volume ratio (Fig. S.1E). ClO_2_^−^ concentration increased gradually with longer polarization times in the tube (Figs. S.5A & S.5B), while remaining almost constant in the hub. The selected operating conditions hold promising potential for treating already existing biofilms in the e-catheter by delivering high concentrations of both HOCl and ClO_2_^−^. However, it also increases the potential risk of introducing toxic concentrations to the bloodstream. As the presence of HOCl (0.5–1 mM) and ClO_2_^−^ (1.5–7.5 mM) at the bloodstream interface may cause hematotoxicity,^71,72^ the solution should be replaced with fresh saline. Another alternative to prevent hematotoxicity is to limit HOCl generation by stopping the polarization of the electrodes before the local concentration reaches hematotoxic levels.

The local pH was observed to be higher in the hub compared to other parts (Fig. S.5D), and pH decreased over time in all compartments (Fig. S.5E). This significant decrease in the pH in all compartments is from the oxidation of Cl^−^ to HOCl and the production of H^+^, as given in Eq. 1, due to more available surface area. At 48 h, the pH in the tube and hub were observed as 1.08 and 2.02, respectively (Fig. S.5F). Although it is not advisable to introduce acidic (pH < 5) solutions into bloodstream, some medications (e.g. vancomycin, doxycycline, dopamine) have an acidic pH (1.8–5). Regardless, having a short CE in the system (only in the hub) limits the surface area available for the hydrogen evolution reaction by reduction of H^+^ into H_2_ according to Eq. 2, which would increase the pH and reduce the accumulation of H^+^ ions.

Manipulating electrode dimensions can modulate the surface area available for electrochemical reactions, thereby influencing HOCl production and distribution in different catheter components. The ability to achieve high concentrations of HOCl within specific regions, such as the Luer lock, holds promising implications for the efficacy of electrochemical catheters in clinical applications. However, high HOCl (>0.5–1 mM)^71^ and ClO_2_^−^ (>1.5–7.5 mM)^72^ concentrations may pose a toxicity risk.^73^ To adjust the concentration distribution, the surface area of the WE can be manipulated.

#### Surface area of the working electrode

**Figure 6.**
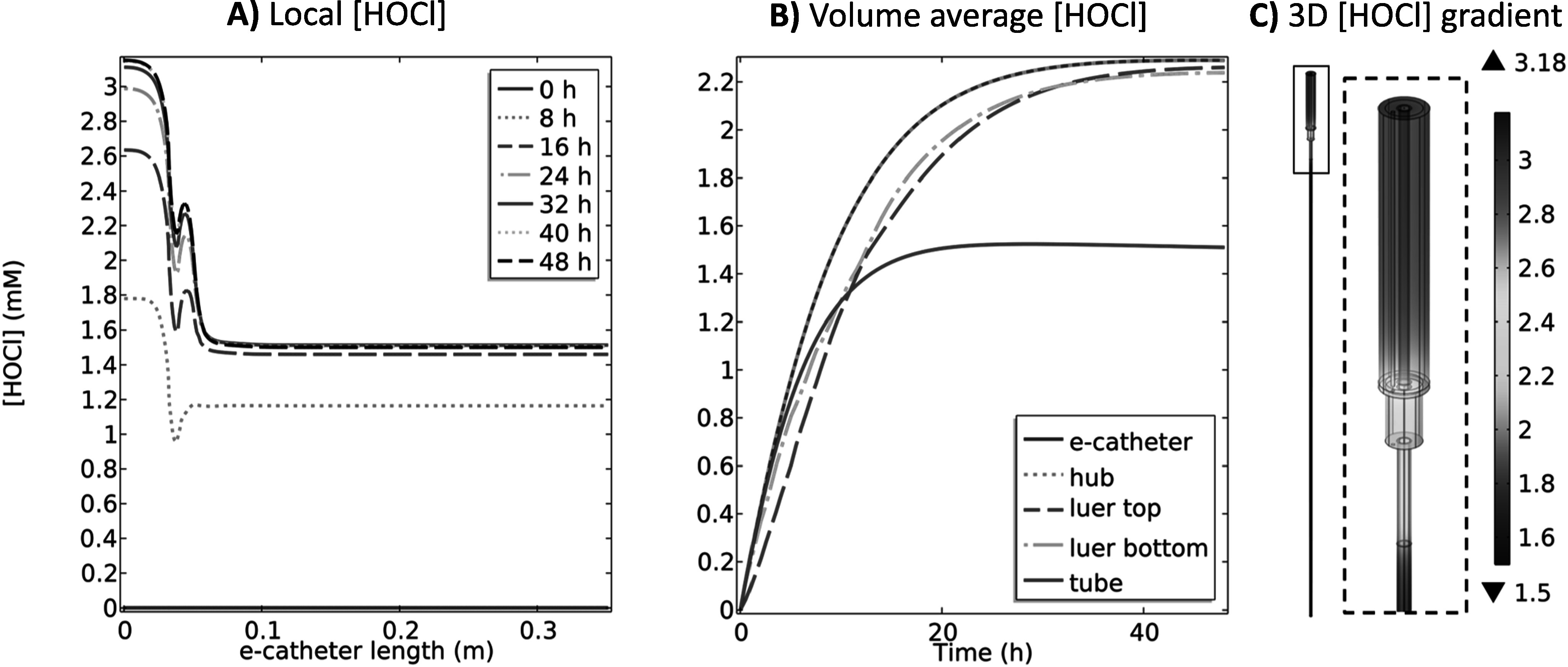
(A) local HOCl (mM) profiles at 0, 8, 16, 24, 32, 40 and 48 h, (B) volume average HOCl (mM) profiles over 48 h and (C) 3D HOCl (mM) gradient at 48 h in the e-catheter, at 1.5 V_Ag/AgCl_ (T_hub_ = 25 °C and T_tube_ = 37 °C) (T_hub_ = 25 °C and T_tube_ = 37 °C) (d_hub,WE_ = 6.15 × 10^–4 ^m, L_hub,WE_ = 3.26 × 10^–2^ m, d_tube,WE_ = 0.54 × 10^–4 ^m and L_tube,WE_ = 0.3 m).

In this simulation, the surface area of the WE in different compartments was adjusted to match HOCl generation in each compartment. The diameter of the WE within the hub region was multiplied by 2.51, while the diameter of the WE within the tube and connector region was divided by 0.22, resulting in the wire having the same surface area/volume (∼2.62 1/m) in the hub and tube regions. The HOCl concentration profile throughout the e-catheter was monitored (Fig. 6) under constant potential (1.5 V_Ag/AgCl_) conditions for 48 h.

Figure 6A shows that local HOCl concentrations peaked in the hub and at the bottom of the Luer lock. Figure 6B demonstrates a notable increase in volume average HOCl concentration in the hub when the WE diameter varied between compartments, compared to when the WE diameter was kept constant (Fig. 5B). HOCl concentration on the WE rose to ∼3.18 mM (Fig. 6C) at 48 h, higher than in the previous observation when the WE diameter was kept constant (Fig. 5C). This increase suggests that the adjusting WE diameter within the hub region will lead to enhanced HOCl production and accumulation, likely due to increased surface area for electrochemical reactions. Conversely, in the tube region, the concentration of HOCl on the WE decreased to ∼1.5 mM (Fig. 6C) at 48 h from ∼4.27 mM (Fig. 5C). This reduction indicates that adjusting the WE diameter within the tube region will result in decreased HOCl production. Decreasing the WE diameter reduces the available surface area for electrochemical reactions, leading to the observed decrease in HOCl concentration within the tube. The volume average HOCl concentrations in all compartments were similar for the first 8 h, while thereafter and through 48 h, HOCl concentrations increased in the hub and Luer lock, and decreased in the tube (Fig. 6B). Similarly, O_2_ and ClO_2_^−^ concentrations increased in the hub and decreased slightly in the tube (Fig. S.1F) compared to the constant diameter observations (Fig. S.1E). Figures S.6A & S.6B show that pH remained almost constant across the whole e-catheter while ClO_2_^−^ concentrations peaked in the hub and the bottom of the Luer lock (Figs. S.6D & S.6E), similar to the HOCl concentrations. Lower HOCl concentration in the tube compared to the hub is due to having a short CE in the system (only in the hub), which helps maintain pH (Fig. S.1F) by the hydrogen evolution reaction according to Eq. 2. Therefore, HOCl and ClO_2_^−^ concentrations progressively increased in the hub and connector with the help of the CE reaction even after 8 h, unlike in the tube.

These results highlight how the active electrode surface area affects HOCl concentration profiles throughout the e-catheter system. Varied electrode surface area can affect the distribution and synthesis of HOCl in different parts of the catheter. In real-life applications, achieving similar HOCl concentrations throughout the e-catheter may involve manipulating the surface area of the wire in the hub and tube regions, which can be achieved by applying a coating on the WE wire, thereby reducing effective surface area rather than physically adjusting the WE wire diameter.

### Strategies to control biocidal efficiency for in vitro testing

The EHAP model can aid in designing in vitro tests for different scenarios; Scenario 1: short WE placed only in the hub (Figs. 2 and 3), Scenario 2: controlling the potential applied to the WE (Fig. 4), Scenario 3: longer WE (Fig. 5), and Scenario 4: longer WE with adjusted surface area (Fig. 6).

In the first scenario, where the electrodes are localized to the hub, the HOCl concentration is highest near the WE, which is located in the hub, and lowest in the tube due to the slow diffusion of HOCl from the hub to the tube. This scenario could be used for infection prevention experiments where the aim is to prevent pathogens from colonizing the hub and spreading to the tube, and eventually the bloodstream.

In the second scenario, the electrodes are also localized to the hub, and the potential applied to the WE manipulated to change HOCl concentrations. This scenario can be used to optimize HOCl production or tailoring production to be higher or lower when needed.

In the third scenario, the WE is present both in the hub and tube while the CE is only present only in the hub. Increasing the WE wire length to match the e-catheter length yields higher HOCl concentrations in the tube compared to the hub. This scenario may would allow for better prevention of colonization of both the hub and tube by generating HOCl throughout the entire catheter. However, it creates risk of toxicity at the bloodstream interface (bottom of the tube).

In the fourth scenario, similar to the third scenario, the WE is present both in the hub and tube, and the CE is only present in the hub. However, in this case, the surface area of the WE is smaller for the tube and higher for the hub part in order to equalize the surface area to volume ratio. The distribution of HOCl concentration can be tuned by changing the WE surface area in different parts of the catheter. Although this approach can be used to produce uniform HOCl concentrations, practically, it is desirable to generate higher concentrations at the entry point at the top of the hub. The advantage of this scenario over scenario three, when the WE diameter is constant, is that the HOCl concentration is no higher in the tube than in the hub, mitigating potential toxicity to a degree.

Regardless of the strategy used, it might be helpful to consider removing the solution prior to infusing through the catheter, to phycially remove any associated biological or chemical byproducts, a strategy used for antibiotic lock practices.

## Conclusions

The aim of this study was to employ numerical modeling to forecast the electrochemical generation of HOCl by an e-catheter. Using the proposed model, simulations were conducted to predict the concentration profiles of electrochemically generated HOCl and the effect of polarization time, diffusion limitation, WE length, and WE surface area on HOCl generation and distribution in the e-catheter. Based on these simulations, the following conclusions can be drawn:

1) When the electrodes are only present in the hub, the highest HOCl concentration (1.72 mM) is on the WE surface in the hub, whereas the lowest concentration (∼0 mM) is at the bottom of the tube after 48 h polarization at 1.5V_Ag/AgCl_. This indicates that HOCl is generated in the hub due to placement of the WE, but that diffusion is limited into the tube during polarization due to the length of the tube (0.3 m). Such a configuration would be effective in preventing pathogen entrance into the hub and pose no risk of generating toxic HOCl concentrations at the bottom of the tube (bloodstream interface).
2) Diffusion of HOCl into the tube (0.3 m) for 120 h after the WE was polarized at 1.5 V_Ag/AgCl_ for 48 h did not alter the HOCl concentrations in the tube, but pH decreased. pH variation is due to diffusion of H^+^, which is faster than that of HOCl due to a larger diffusion coefficient. This strategy also does not pose a risk of introducing toxic HOCl concentrations into the bloodstream.
3) Increasing the potential applied to the WE increased the species transport rate due to increased HOCl generation rates and concentrations. However, the HOCl concentration at the bottom of the tube remained ∼0 mM, minimizing potential toxicity.
4) Increasing the WE length resulted in increased HOCl concentrations in the tube. The maximum HOCl concentration in the tube was recorded as ∼2.7 times higher than the minimum HOCl concentration in the hub when the length of the WE was increased and matched to the catheter length (0.33 m). This concentration effect is due to the ratio of the surface area of the WE to the luminal volume. Such a configuration carries the risk of introducing HOCl into the bloodstream if the system is not carefully controlled.
5) Altering the surface area of the WE in different compartments impacted local production and distribution of HOCl. Increasing surface area in the hub region improved HOCl generation and accumulation, while decreasing surface area in the tube region reduced HOCl concentrations in the tube due to surface area availability of the WE. Manipulating the electrode surface area presents a potential strategy for controlling HOCl distribution throughout the e-catheter.
